# The Impact of the COVID-19 Pandemic on the Physical Fitness of Primary School Students in China Based on the Bronfenbrenner Ecological Theory

**DOI:** 10.3389/fpsyg.2022.896046

**Published:** 2022-06-29

**Authors:** Hailing Li, Jadeera Phaik Geok Cheong

**Affiliations:** ^1^Centre for Sport and Exercise Sciences, Universiti Malaya, Kuala Lumpur, Malaysia; ^2^UM STEM Centre, Universiti Malaya, Kuala Lumpur, Malaysia; ^3^Pusat Kajian Kecemerlangan Melayu, Universiti Malaya, Kuala Lumpur, Malaysia

**Keywords:** children, coronavirus, exercise, fitness assessments, fitness tests, physical education

## Abstract

After the outbreak of the COVID-19 pandemic, nation lockdown became an effective way to isolate the spread of the virus. Schools were postponed, students had to stay at home and opportunities for physical activity amongst school children were severely affected. This research sought to determine the impact of the pandemic on the physical fitness of primary school students. In total, 1,235 students from grades one to five in a primary school in Beijing took part in this research. Using the Chinese National Student Physical Fitness Standard as a guide, the students were subjected to BMI, vital capacity, 50 m sprint, sit and reach, timed rope-skipping, timed sit-ups, and 50m × 8 shuttle run measurements. These tests were administered once before and once after the lockdown period. The results showed that the overall physical fitness of the participants was better after the lockdown [*p* = 0.000, r = −0.14, 95% CI (–0.219, –0.061)]. Specifically, vital capacity, sit and reach, timed rope-skipping and timed sit-ups had improved after the lockdown. Meanwhile, 50m × 8 shuttle run dropped slightly but not significantly whereas 50 m sprint dropped sharply after the lockdown. The proportion of overweight and obese students increased, but the difference before and after the lockdown was small. It appeared that during the pandemic, through the intervention of many comprehensive factors, home-based fitness was normalized and promoted the healthy development of students.

## Introduction

In late 2019, the emergence of a novel human coronavirus, Severe Acute Respiratory Syndrome Coronavirus 2 (SARS-CoV-2), triggered a global COVID-19 pandemic (Schmidt et al., 2020) and posed a health threat of unknown magnitude worldwide (Chen et al., 2020a). Due to the easy spread of the virus and the emergence of new variants, no effective medication has been found to eliminate the virus completely. Instead, lockdown was an effective way to interrupt the transmission chain and control the pandemic. Lockdown was first implemented in Wuhan on January 23, 2020, in China. As the epidemic continued, all provinces, municipalities, and autonomous regions had activated the Level I response to major public health emergencies on January 29, 2020 (IFENG, 2020), which is the highest level of response to major public health emergencies (The Central People’s Government of the People’s Republic of China, 2006; IFENG, 2020). Lockdown or strict control measures were implemented across the country, which included imposing a lockdown on the infected area, restricting or stopping fairs, gatherings, and theater performances, shutting down work and businesses, and implementing closed community management, with residents disallowed to leave their homes, and home services were provided, including home delivery and home treatment by medical staff (The Central People’s Government of the People’s Republic of China, 2006). The lockdown was not lifted until the regions downgraded their Level I response to Level II or more lower-level accordance with the public health emergency level criteria based on the control of the epidemic (Tianjin Daily, 2020). In terms of schools, for the safety of students and to prevent the spread of the virus on campus grounds, and the February 19–June 16, 2020, semester was scheduled to home-based online classes (Minstry of Education of the People’s Republic of China, 2020a). As the outbreak was brought under control, students were able to re-enter the classroom for the new semester on September 1, 2020, and resume their regular studies.

In facing the COVID-19 pandemic, what impact did the lockdown have on students’ health? On the one hand, students could not go out of the house freely and could not walk to the park or playground for exercise, which reduced the time for physical activities but increased the screen time for watching TV or playing with cell phones, which will inevitably bring adverse effects to students’ health (Carroll et al., 2020; Cuschieri and Grech, 2020; Pietrobelli et al., 2020). In addition, for active students, staying at home for a long time might affect students’ mental health and cause problems such as lonely, restless, anxiety, or uneasy (Adıbelli and Sümen, 2020; Duan et al., 2020; Francisco et al., 2020). As physical fitness was considered a powerful marker of health in children (Ortega et al., 2008), that will subsequently determine health in adulthood (Malina, 2001; Ding and Jiang, 2020), it is growing in significance to everyday life (American College of Sports Medicine, 2013). Physical fitness was reported to be essential for performing school assignments and meeting home responsibilities, with enough energy for sport and alternative leisure activities (Corbin et al., 2014). Furthermore, fitness can help build a stronger immune system to guard against viral and bacterial infections (Corbin et al., 2012). On the contrary, low fitness level was associated with negatively impacting health outcomes, such as obesity, heart disease, impaired skeletal health, and poor quality of life (De Miguel-Etayo et al., 2014). Physical fitness was found to be affected by genetic factors, but also by physical activities (Bouchard et al., 2012; Takken and Hulzebos, 2013), and only regular physical activity could achieve optimal physical fitness (Corbin et al., 2012). There were also studies showing that maintaining regular physical activity habits was a key strategy for mental health, which could help regulate mood (Kaur et al., 2020; Maugeri et al., 2020). Therefore, during the special period, it was very necessary to continue to carry out physical activities at home.

**FIGURE 1:**
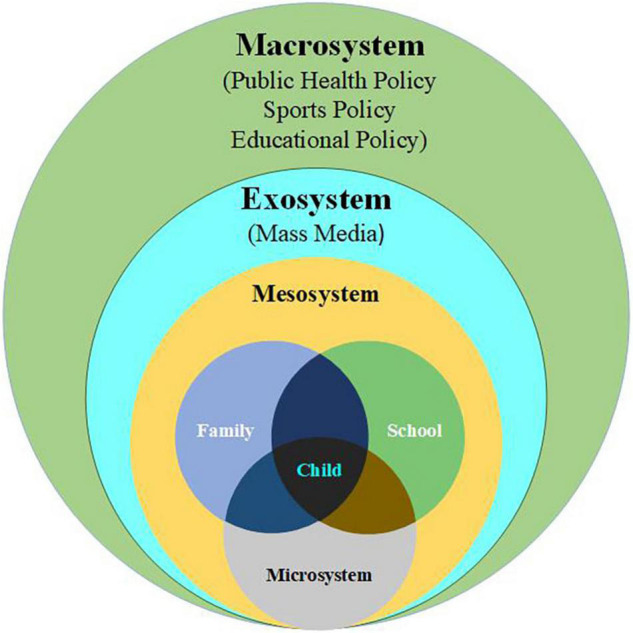
Bronfenbrenner’s ecological model. Image reproduced with minor modifications from Bennett and Grimley (2001), with permission from Elsevier (License number 5334520439220).

Therefore, this study sought to determine the rank distribution of physical fitness indicators of primary school children and to compare the fitness levels of students before and after the lockdown, The interpretation of the results was conducted according to Bronfenbrenner (1977) ecological theory, which explained that throughout the life span, an individual was embedded in multiple environments, such as the family, school, and social environments. Figure 1 outlines the different environmental factors in each level of the Ecological Theory Framework, providing a useful theoretical framework that focuses on the major factors affecting children’s development (Bronfenbrenner, 1979; Sallis et al., 2008; Browne et al., 2021; Lopez et al., 2021; Teran-Escobar et al., 2021; Stienwandt et al., 2022). The findings of this study will provide a reference value for similar public health emergencies in the future.

## Materials and Methods

### Participants

A primary school in Beijing, China, was invited to participate in the study. Prior to the lockdown, 1,428 children in this school underwent the physical fitness assessments. Following the lockdown, a total of 1,235 students again underwent the physical fitness assessments, and were included in the final sample (638 boys and 597 girls). The remaining 193 children whose evaluation were previously recorded, were promoted to middle school and were excluded from the study. All students read the Participant Information Form, and their parents or guardian signed the informed consent form. This study was conducted according to the procedures as approved by the University of Malaya Research Ethics Committee (UM.TNC2/UMREC – 667).

### Procedures

There were two data collection sessions, one before lockdown (October 2019) and one after lockdown (October 2020). All CNSPFS tests were administered during normal school hours according to standard operating protocols by five trained physical education (PE) teachers.

Before the assessment, the researcher explained each test and demonstrated each test technique. Participants were allowed one practice trial for each test. The researchers tested the subjects in accordance with the test protocol and requirements. At the end of all tests, direct scores were obtained for each of the seven tests and raw data were input into a central spreadsheet.

### Measures

The most current fitness assessment requirements and standards in China, the 2014 revised Chinese National Student Physical Fitness Standard (CNSPFS) battery (Ministry of Education of the People’s Republic of China, 2014), was used to assess the physical fitness of participants. The CNSPFS was tailor-made for children of different grades, that is, different test standards were used for different grades and genders (Zhu et al., 2017). The testing battery is a common testing battery that has been widely used to evaluate children’s physical fitness in China.

This battery consisted of a standardized test, that involved a total of seven fitness indicators, which are Body Mass Index (BMI), vital capacity (VC) of the lung, 50 m sprint, sit and reach, timed rope-skipping, timed sit-ups, and 50 m × 8 shuttle run (Zhu et al., 2017), covering areas of body mass index, aerobic capacity, speed, flexibility, and abdominal strength. Children’s overall physical fitness performance was categorized, per CNSPFS standards, as excellent, good, pass, or fail (Ministry of Education of the People’s Republic of China, 2014).

#### BMI

A portable instrument (GMCS-IV; Jianmin, Beijing, China) was used to measure the height and weight of the students and to obtain the BMI, which was calculated by taking a person’s weight and dividing by their height squared. During testing, the subject stood on the bottom plate of the equipment with bare feet, back to the upright posture, with the head upright, the torso naturally straight, the upper limbs naturally drooping, the heels close together, and the toes 60 degrees apart. After 2–3 s, the measurement result was displayed on the LCD, and the voice broadcast prompt was synchronized. All participating children from Grades 1–6 were assessed for this measure.

#### Vital Capacity

The vital capacity (VC) was assessed by using a spirometer (GMCS-IV; Jianmin, Beijing, China), which is a device that measures the maximum amount of air that can be fully exhaled from the lung after a maximum inhalation. During the test, the participant stood in front of the instrument, installed the disposable mouthpiece on the side of the air inlet push tube, and held the handle of the instrument firmly. Participants were asked to bring the mouthpiece close to their mouth, take a deep breath, and blow at a moderately uniform speed until they stopped blowing. Participants were not allowed to stop breathing during the process or inhale twice during the test. Each participant was tested twice and the maximum value taken. All participating children from Grades 1–6 were assessed for this measure.

#### 50 m Sprint

To assess speed in this test, in addition to the 50-m straight race track, a starting flag, a whistle and a stopwatch were also needed. Participant started from a stationary standing position with one foot in front of the other and the front foot must be behind the starting line. Once the participant was ready and motionless, the starter gave the instructions “set” then blew whistle and waved the starting flag to provide the signal to the finish line timekeeper to start timing. After hearing the whistle, participants had to run across the finish line at the fastest speed. The timekeeper stopped timing at the same time. Two trials were allowed, and the best time was recorded to the nearest two decimal places. All participating children from Grades 1–6 were assessed for this measure.

#### Sit and Reach

Flexibility was measured using the sit and reach test which was carried out by a seat forward flexion tester (GMCS-IV; Jianmin, Beijing, China). During the test, the participant sat on the flat ground with legs straight and legs flat against the test longitudinal plate. The legs were about 10–15 cm apart. The upper body was bent forward. Participants took the test twice with the best score recorded in centimeters (cm), to the nearest one decimal place. All participating children from Grades 1–6 were assessed for this measure.

#### Timed Rope-Skipping

To test children’s coordination and muscle endurance, a rope-skipping test was conducted requiring them to land on both feet after they got off their feet. Participants were instructed to skip continuously for 1 min after they were given the appropriate length of ropes. The tester counted and recorded the number of rope-skipping. All participating children from Grades 1–6 were assessed for this measure.

#### Timed Sit-Up

Abdominal muscle strength and endurance was measured using a 1-min sit-up test. Participants were divided into pairs. One participant lied on their back with their legs slightly apart, their knees bent at a 90-degree angle, and held the head with hands close to ears while the other partner held down the ankle joint to secure the lower limb. During the performance, participants had to elevate their trunks to the point where the elbows made contact with their thighs, then lowered their shoulders back to the mat to resume the starting position. When given the “start” signal, the tester began the stopwatch and recorded the number of times of completed sit-ups within 1 min. This measure was assessed only for primary school children from Grades 3–6.

#### 50m × 8 Shuttle Run

Cardiovascular endurance was tested using 50 m × 8 shuttle run test. During this test, participants needed to run back and forth eight times along a straight track line between 2 poles set 50 m apart. Participants were asked to run at their maximum speed and, at the end of the track line, turn around at a pole in a counter- clockwise direction, and run back to the starting line. A stopwatch was used in the test, and time was recorded to the nearest second. This measure was assessed only for primary school children from Grades 5–6.

### Data Analyses

The raw results of all tests were first calculated using grade- and sex-specific weights defined by the 2014 revised CNSPFS (Ministry of Education of the People’s Republic of China, 2014), and weighted scores were subsequently categorized into the categories of excellent (defined as having scores of 90), good (scores 80–89), pass (scores 60–79), or fail (scores <60).

Descriptive statistics were used to analyze all tests, with means and standard deviations (SD) reported. Moreover, the scores ranked distribution [n (%)] were reported for each PF outcome to compare the number of the subjects who fell within the established standards of the test items. McNemar–Bowker Test was used to test for differences in the distribution between the two time points. Kolmogorov–Smirnov test was used to identify normality, and all assumptions for Non-parametric Wilcoxon test were met when making before and after lockdown comparisons. An effect size, r, was calculated using the conversion formula (Morgan et al., 2019), with effect size > 0.5 considered as large, >0.3 as medium, and >0.1 as small (Cohen, 1988).

Statistical significance was set at *p* < 0.05, and all analyses were performed using SPSS Statistics for Windows version 25.0.

## Results

### Rank Distribution of BMI and Physical Fitness

For BMI rank distribution, overweight and obese students increased after the lockdown compared to before the lockdown. Obesity increased by 0.8% while overweight increased by 2.6% between the two time periods.

Correspondingly, underweight and normal students reduced after the lockdown compared to below the lockdown, Underweight reduced by 1.2% while normal reduced by 2.2% between the two time periods. The breakdown for BMI rank distribution before and after the lockdown are shown in Table 1.

**TABLE 1:** BMI rank distribution.

Indicator	Year	Underweight	Normal	Overweight	Obesity
		(*n* and %)	(*n* and %)	(*n* and %)	(*n* and %)
BMI	2019	46 (3.7)	913 (73.9)	159 (12.9)	117 (9.5)
BMI	2020	31 (2.5)	885 (71.7)	192 (15.5)	127 (10.3)

**FIGURE 2:**
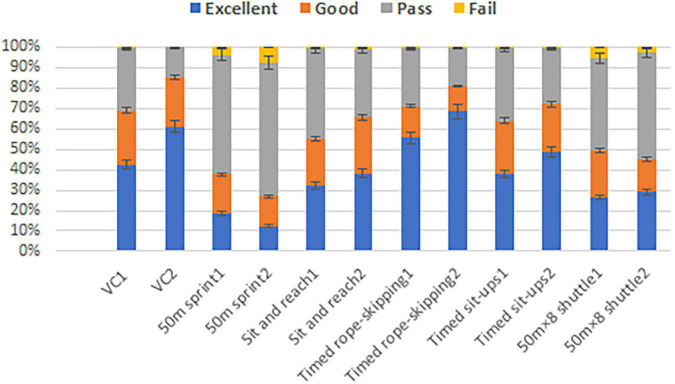
Physical fitness rank distribution before and after the lockdown. 1-2019; 2-2020.

For Physical Fitness indicators, Table 2 and Figure 2 provide information about the changes in the rank distribution before and after the lockdown. The results show that VC, timed rope-skipping and timed sit-ups indicators had all increased in the proportions of excellent rank, while the proportions of other ranks had all decreased after the lockdown. In terms of sit and reach, although the proportions of excellent and good rank had also increased, the fail rate had also increased after the lockdown. Similarly, the comparison results of the 50 m × 8 shuttle run index showed that the proportions of excellent had increased, however, the proportions of good and fail ranks had decreased after the lockdown. Conversely, for the 50 m sprint, the indicator showed that the proportions of excellent and good ranks had decreased, while the proportion of pass and fail ranks had increased after the lockdown.

**TABLE 2:** The physical fitness rank distribution.

Indicator	Year	Excellent	Good	Pass	Fail	Sum P&F (*n* and %)
		(*n* and %)	(*n* and %)	(*n* and %)	(*n* and %)	
VC	2019	525 (42.51)	325 (26.32)	383 (31.01)	2 (0.16)	385 (31.17)
	2020	754 (61.05)	295 (23.89)	186 (15.06)	0 (0.00)	186 (15.06)
50m sprint	2019	232 (18.79)	232 (18.79)	722 (58.46)	49 (3.97)	771 (62.43)
	2020	154 (12.47)	180 (14.57)	804 (65.10)	97 (7.85)	901 (72.95)
Sit and reach	2019	396 (32.06)	287 (23.24)	542 (43.89)	10 (0.81)	552 (44.7)
	2020	472 (38.22)	339 (27.45)	406 (32.87)	18 (1.46)	424 (34.33)
Timed rope-skipping	2019	687 (55.63)	192 (15.55)	350 (28.34)	6 (0.49)	356 (28.83)
	2020	846 (68.50)	153 (12.39)	233 (18.87)	3 (0.24)	236 (19.11)
Timed sit-ups	2019	256 (38.04)	175 (26.00)	239 (35.51)	3 (0.45)	242 (35.96)
	2020	326 (48.44)	158 (23.48)	187 (27.79)	2 (0.30)	189 (28.09)
50m×8 shuttle run	2019	67 (26.48)	58 (22.92)	114 (45.06)	14 (5.53)	128 (50.59)
	2020	74 (29.25)	40 (15.81)	132 (52.17)	7 (2.77)	139 (54.94)

*Sum P&F means summary of Pass and Fail.*

### Comparison of Scores Before and After Lockdown

Normality of data was assessed using the Kolmogorov–Smirnov test. It was revealed that the data for all seven test indicators were not normally distributed (*p* < 0.05). Subsequently, the Wilcoxon signed ranks test, a non-parametric paired difference test, was selected to compare the performance of students before and after lockdown.

As shown in Table 3, the total results of the participants show that 41.13% of the 1235 students had lower scores after the lockdown, with the greatest decrease in the scores of 50 m and 50 m × 8 shuttle run, corresponding to 58.14 and 47.43%; 51.50% of the students had better results after the lockdown; 7.37% of the students had no changes. This difference indicated that the physical fitness of the students had significantly increased to a certain extent after the lockdown, with *z* = −4.743, *p* = 0.000, *r* = −0.14, a small effect size according to Cohen (1988).

**TABLE 3:** Wilcoxon signed ranks test for all CNSPFS tests.

Indicator	Before	After	Rank score (after-before)				Z	Asymp. Sig. (2-tailed)	R	95%CI
	Mean ± SD	Mean ± SD	Negative ranks (%)	Positive ranks (%)	Ties (%)	N				
Total score	84.21 ± 7.50	84.95 ± 7.90	41.13	51.50	7.37	1,235	−4.743	0.000	−0.14	(–0.219, –0.061)
BMI	92.89 ± 12.95	92.28 ± 13.31	12.47	9.23	78.30	1,235	−2.118	0.034	−0.06	(–0.139, 0.019)
VC	85.35 ± 11.17	90.67 ± 10.06	18.54	53.20	28.26	1,235	−16.117	0.000	−0.46	(–0.540, –0.380)
50 m sprint	76.21 ± 16.07	72.13 ± 17.43	58.14	26.72	15.14	1,235	−12.209	0.000	−0.35	(–0.429, –0.271)
Sit and reach	81.95 ± 11.62	83.75 ± 13.03	27.13	46.80	26.07	1,235	−8.456	0.000	−0.24	(–0.319, –0.161)
Timed rope-skipping	88.11 ± 12.49	91.43 ± 11.12	23.97	40.81	35.22	1,235	−9.658	0.000	−0.28	(–0.359, –0.201)
Timed sit-ups	84.64 ± 11.26	86.86 ± 11.27	29.57	45.62	24.81	673	−5.339	0.000	−0.21	(–0.317, –0.103)
50 m × 8 shuttles run	78.60 ± 16.19	78.49 ± 14.92	47.43	39.13	13.44	253	−0.656	0.512	−0.04	(–0.214, 0.134)

With the exception of 50 m × 8 shuttle run, the difference between the scores before and after the lockdown for BMI, VC, 50 m sprint, sit and reach, timed rope-skipping, and timed sit-up were all statistically significant. BMI (z = −2.118, *p* = 0.034, *r* = −0.06) and 50m sprint (z = −12.209, *p* = 0.000, *r* = −0.35) had decreased, with small and medium effect sizes respectively. Conversely, timed sit-ups (*z* = −5.339, *p* = 0.000, *r* = −0.21) had increased with a small to medium effect size, both sit and reach (*z* = −8.456, *p* = 0.000, *r* = −0.24) and timed rope skipping (*z* = −9.658, *p* = 0.000, *r* = −0.28) increased with small to medium effect sizes. VC (*z* = −16.117, *p* = 0.000, *r* = −0.46) had increased with medium to large effect sizes. Scores of 50 m × 8 shuttle run before and after the lockdown were not significantly different, *z* = −0.656, *p* = 0.512 > 0.05, *r* = −0.04, and decreased with a small effect size.

## Discussion

This study sought to determine the rank distribution of physical fitness indicators of primary school children and to compare their fitness levels before and after the lockdown. For rank distribution, the proportion of overweight and obese students increased after the lockdown compared to before the lockdown. As for the remaining physical fitness indicators, except for the 50 m sprint, all other indicators had increased in proportions of excellent rank, with a majority accompanied by decreased in fail ranks. Notably, the 50 m sprint had decreased in the excellent and good ranks, and increased in the pass and failure ranks. As for comparisons between the physical fitness scores, the overall results after the lockdown were better than those before the lockdown. The results of the specific tests show that, except for BMI, 50 m sprint, and 50 m × 8 shuttle run scores, other test results had improved. Among the decreasing indicators, the difference in BMI before and after was statistically significant, but the magnitude was small. As for the 50 m × 8 shuttle run, there was a drop but it was not statistically significant. Only the 50 m sprint had a large significant decline. It appeared that the negative impact of the lockdown period on the physical fitness of the students was mainly concentrated on the speed quality of running.

The results of this study indicated that there were improvements in some physical fitness indicators, in contrast to previous studies (Ito et al., 2021; Pombo et al., 2021; Jarnig et al., 2022), in which the results showed that the lockdown had negative impact on the physical fitness of the children. In the longitudinal study of the impact of mitigation measures concerning COVID-19 on the health and well-being of primary school students, it was found that the closures of schools and sports facilities resulted in the adverse combination of increased BMI and decreased physical fitness (Jarnig et al., 2022). Pombo et al. (2021) reported that less physical activity, a sedentary lifestyle, and more screen time were the reasons for the decline in physical fitness after the lockdown. To explain why this study found significant increases instead of decreases in physical fitness after lockdown, the Bronfenbrenner Ecology Theory appeared to be a probable and suitable model to be used for the main points of discussion. According to the Bronfenbrenner Ecology Theory, macrosystems include the public health system and educational policy (Lenhoff and Pogodzinski, 2018), which influence child development primarily through their association with family-and school-level factors (Su, 2012).

### Macrosystem Level

The first-level public health emergency response was activated across China following the outbreak of the COVID-19 pandemic in December 2019 due to the strong spread, unpredictability, and serious harm of the COVID-19. Lockdown policy was implemented, large-scale events had been cancelled or postponed, opening of schools were postponed, and people had reduced their outings to stay at home to minimize cross-infection and cut off the spread of the virus to the greatest extent (Li and Liu, 2020; Liu et al., 2020). Staying at home for a long time without going out, sitting for a long time, playing with mobile phones, and lying down for a long time had become a living condition for most people during the pandemic prevention and control period (Zhuang, 2020). In the face of this situation, on January 30, 2020, the General Administration of Sport of China issued a notice on promoting scientific home-based fitness methods, requiring local sports departments to introduce simple, scientific, and effective home-based fitness methods based on local conditions to guide people to start a healthy home-based lifestyle (General Office of the General Administration of Sport of China, 2020). Later on February 12 and 16, the Ministry of Education of the People’s Republic of China issued a Notice on Work Arrangements titled “Suspension of Classes without Suspension of Learning” during the postponement of the start of primary and secondary schools and another Notice for parents of primary and middle school students in China as a guide for children’s home study and life during the pandemic, requiring schools and parents to guide students to arrange indoor physical exercises to enhance students’ physical fitness and ensure maintenance of their health (Minstry of Education of the Peop’s Republic of China, 2020b; Minstry of Education of the People’s Republic of China, 2020a). These policies had indirectly promoted the health of children.

### Exosystem Level

At the Exosystem level, the mass media became an essential tool for combating COVID-19, which offered a unified platform for all public health communications, comprehensive healthcare education guidelines, and robust social distancing strategies while still maintaining social connections (Anwar et al., 2020).

Various information about “home-based fitness” was disseminated on social media platforms, including informational and motivational persuasive sports health information (Huang et al., 2020). For example, General Administration of Sport of China (General Office of the General Administration of Sport of China, 2020), the relevant local sports departments used the Internet, TV, radio, and other media resources to widely publicize the importance of home fitness, and made people more aware of the benefits of physical exercise for physical health, that proper physical exercise can not only improve physical functions but also enhance immunity (Nieman, 2000; Leandro et al., 2007; da Silveira et al., 2021). Social media also provides sports health prescriptions, cloud competitions, and cloud activities organization and planning which had greatly stimulated the participation and creativity of sports participants in the “cloud” and promoted national fitness through competitions, punch cards, and relays (Huang et al., 2020).

In addition, the most important impact on children was mass media, such as DingTalk and MOOC, which provided online educational videos (Guo et al., 2014; Ai and Huang, 2021), had maintained the original teaching plan disrupted by the pandemic, and restored normal teaching order by providing online education platforms for schools and families (T. Chen et al., 2020b).

### Mesosystem and Microsystem Level

For children, whether the school and family setting at Microsystem level, or the relationship between family and school at the Mesosystem level, all these relationships affected children’s health and played the most direct role in helping them cope with the pandemic.

In order to make up for the lack of exercise time and promote the normal growth and development of children during the pandemic, the Ministry of Education of the People’s Republic of China had proposed that “Suspension of Class without Suspension of Learning” (Minstry of Education of the People’s Republic of China, 2020a). Hence all schools practiced online guidance to allow students to learn and participate in physical exercises at home, and conveyed through the network platform, home-based physical exercise videos for students to watch and learn, and improve the effectiveness of their exercise. At the same time, the schools had established an effective evaluation and monitoring system by using software such as DingTalk’s “Physical Fitness Training Every Day”, and WeChat to perform physical check-in tasks for students (Xiang et al., 2020).

On the family side, parents began to understand the value of exercise, which is that regular exercise of sufficient intensity could be used as an auxiliary tool to strengthen and prepare the immune system for COVID-19 (da Silveira et al., 2021). Parents encouraged their child to participate in more sports activities (De La Haye et al., 2014) and increased the consumption of exercise equipment which affected children’s physical activity behaviors (Sirard et al., 2010). For example, at the beginning of the COVID-19 outbreak, sales of treadmills increased by 173.3% on a year-on-year ratio.

In the context of school and family relationships, home-school cooperation had become an important force to ensure the effectiveness of online physical education (Shu and Xia, 2020). As professionals, PE teachers provided parents with professional education guidance through online videos and articles related to the value, function, content, methods, and experience of home parent-child exercise. This was carried out with the help of online platforms such as class QQ groups and WeChat groups. Parents also provided in-PE learning assistance and after-PE exercise feedback for PE teachers’ online teaching. Through the interaction between teachers and parents, an educational synergy was finally formed, which played a positive role in the growth and development of students (Hu et al., 2020; Shu and Xia, 2020).

Using the Bronfenbrenner Ecological Theory as the focus of discussion, it was possible to conclude that the overall physical fitness of the students before and after the lockdown showed an upward trend, which was achieved through the various efforts of the public health policies, mass media, parents, and schools. However, due to the objective reality of home exercise, running activities for endurance and speed have shown a downward trend. The main reason was that home-based exercise venues were mostly in narrow venues such as living rooms, bedrooms, and corridors, and the scope of activities related to running endurance and speed was limited. It appeared that the choices of home-based exercise programs for children were possible mainly for the development of strength, flexibility, coordination rather than speed and endurance (Xu, 2020; Zagalaz-Sánchez et al., 2021). Future research should investigate how to improve sprint performance and endurance in the home environment. As for the changes of BMI scores, studies had shown that the lockdown had led to unhealthy nutrition in some people, such as excessive intake of snacks and sweets, and eating in response to boredom which easily led to conditions of overweight or obesity (Rundle et al., 2020; Deschasaux-Tanguy et al., 2021; Jenssen et al., 2021). However, due to home-based fitness which helped students to consume parts of energy and get rid of fat production, there was only a small difference in the status of overweight and obesity before and after the lockdown. A limitation of this study was the lack of indicators such as students’ daily exercise time and intensity, and parents’ ages, parents’ educational level, and parents’ exercise habits, which might impact students’ physical fitness results (Rossi et al., 2021).

## Conclusion

The results of this study showed that students’ physical health was effectively intervened during the lockdown period. Under the combination of efforts at all levels of the ecological model to guide the students to include home-based fitness into everyday life and to promote students’ individual health behavior, students’ physical health was maintained during the pandemic.

## Data Availability Statement

The raw data supporting the conclusions of this article will be made available by the authors, without undue reservation.

## Ethics Statement

The studies involving human participants were reviewed and approved by University of Malaya Research Ethics Committee. Written informed consent to participate in this study was provided by the participants’ legal guardian/next of kin.

## Author Contributions

HL conceptualized the research, collected and analyzed data, and drafted the manuscript. JC supervised the research, interpreted the data, and reviewed the manuscript. Both authors contributed to the article and approved the submitted version.

## Conflict of Interest

The authors declare that the research was conducted in the absence of any commercial or financial relationships that could be construed as a potential conflict of interest.

## Publisher’s Note

All claims expressed in this article are solely those of the authors and do not necessarily represent those of their affiliated organizations, or those of the publisher, the editors and the reviewers. Any product that may be evaluated in this article, or claim that may be made by its manufacturer, is not guaranteed or endorsed by the publisher.
